# From Fear to Adaptation: The Journey of Patients with Liver Cancer Living with the Fear of Cancer Recurrence

**DOI:** 10.3390/curroncol32120687

**Published:** 2025-12-04

**Authors:** Eunjin Jo, Ka Ryeong Bae

**Affiliations:** 1 Department of Nursing, Kyungnam University, Changwon 51767, Republic of Korea; eunjinjo@kyungnam.ac.kr; 2 Department of Clinical Research Design and Evaluation, SAIHST, Sungkyunkwan University, Seoul 06355, Republic of Korea

**Keywords:** cancer survivors, hepatocellular carcinoma, fear, recurrence, oncology qualitative research

## Abstract

The study aimed to understand how patients with liver cancer experience and adapt to the fear of cancer recurrence, providing insights into psychological processes and strategies that can inform psycho-oncology research and interventions. In-depth interviews were conducted with 13 patients with liver cancer from December 2019 to February 2020 and analyzed using Colaizzi’s phenomenological method. Four theme clusters emerged: (1) “Inevitable reality of recurrence,” which highlighted the acceptance of recurrence; (2) “Amplified fears,” which reflected heightened emotional distress; (3) “Changes in daily life driven by fear,” which illustrated lifestyle changes driven by uncertainty; and (4) “Living with fear,” which described adaptive strategies and resilience. The findings highlight the need for targeted psycho-oncological approaches to address the fear of cancer recurrence in patients with liver cancer, supporting the development of resilience and enhancing their overall quality of life. Further research is essential to design tailored strategies that reduce psychological distress and promote long-term survivorship.

## Simple Summary

For people with liver cancer, the fear that their illness may return is a powerful psychological burden that influences emotions, social relationships, and daily life. This study explored how patients experience and adapt to this fear by carefully examining their personal accounts. We found that many patients accepted recurrence as an unavoidable possibility, yet still struggled with intense anxiety and uncertainty. These fears often shaped lifestyle choices and everyday routines, while some individuals developed strategies to adapt and maintain resilience. By revealing the inner experiences and coping mechanisms of patients, this study deepens our understanding of how fear of recurrence affects psychological well-being and survivorship. The findings provide insights that can guide future psycho-oncology research and the development of interventions aimed at reducing distress, strengthening adaptive coping, and ultimately supporting better quality of life for people living with and beyond liver cancer.

## 1. Introduction

### 1.1. Background

Primary liver cancer is the sixth most common cancer globally and the third leading cause of cancer-related mortality [1]. In South Korea, liver cancer ranked seventh in prevalence and second in mortality, with 20.6 deaths per 100,000 population in 2020, making it the leading cause of cancer-related deaths among individuals aged 40–59 years [2]. Additionally, liver cancer imposes the highest economic burden among all cancers in South Korea, amounting to USD 2.27 billion annually [3]. Hepatocellular carcinoma (HCC) accounts for 90% of primary liver cancer cases, with most patients also diagnosed with chronic hepatitis or liver cirrhosis. Despite treatment, recurrence rates remain high [2,4].

Fear of cancer recurrence (FCR), defined as the fear that cancer might return or progress, is one of the most common and distressing concerns for cancer survivors [5,6]. Although closely related, fear of cancer recurrence and fear of cancer progression have been suggested to represent overlapping yet conceptually distinct constructs, particularly among patients with confirmed disease activity [7]. While low levels of FCR can encourage adaptive behaviors, such as health monitoring, higher levels are linked to emotional distress, increased healthcare utilization, and reduced quality of life [8,9,10]. Although research on FCR has focused on breast and ovarian cancers, the unique challenges faced by patients with liver cancer—characterized by high recurrence rates—remain underexplored. Compared to breast and gynecological cancers, hepatocellular carcinoma is associated with higher recurrence rates and less favorable long-term outcomes, which may intensify the psychological burden related to fear of recurrence [11,12]. Mixed cancer-type studies often obscure the distinct psychological impacts of high-recurrence cancers like liver cancer [13,14]. This research gap highlights the urgent need to guide future psycho-oncology research and interventions that address the psychological burden of FCR in patients with liver cancer and improve their quality of life.

Furthermore, in the Korean sociocultural context, FCR among patients with HCC may be uniquely intensified by stigma and social expectations surrounding liver disease. Liver cancer and alcohol-related liver conditions are often perceived through a lens of personal responsibility, contributing to stigma, delayed help-seeking, and heightened psychological distress among affected individuals [15,16]. This stigma may foster self-blame, shame, and social withdrawal, further amplifying the emotional burden associated with recurrence risk.

Additionally, Korea’s workplace and social culture are deeply intertwined with alcohol-centered gatherings, where after-work drinking (hoesik) functions as a key mechanism for relationship-building and organizational cohesion. However, these practices can place psychological strain on individuals with liver disease, who may feel conflicted between social expectations and health-related limitations [17,18]. The combination of disease-related stigma and culturally embedded drinking norms may uniquely shape and intensify the experience of FCR among Korean patients with HCC, underscoring the need for culturally informed psycho-oncology research.

Existing qualitative studies on liver cancer have primarily examined illness experiences, quality of life, participation in recovery programs, terminal illness, and treatment management [19,20,21,22,23,24]. However, research on the psychological experiences of patients with liver cancer is limited, restricting a comprehensive understanding of their FCR-related challenges.

### 1.2. Purpose of the Study

This study aims to explore the meaning and structure of FCR experiences in patients with liver cancer, providing an in-depth understanding of how these experiences shape their psychological well-being, social interactions, and daily lives. Using Colaizzi’s phenomenological method, the study examines how patients with liver cancer perceive, internalize, and cope with FCR.

The primary research question is as follows: What are the experiences of patients with liver cancer regarding FCR? The findings aim to inform the development of tailored psychological support strategies to address FCR, improving the quality of life and health management behaviors of patients with liver cancer.

## 2. Methods

### 2.1. Participants and Ethical Considerations

This study targeted patients with liver cancer aged 19 or older who routinely visited the outpatient department after completing active treatment at a tertiary university hospital in South Korea. Recruitment occurred between December 2019 and February 2020 through posted notices in the liver cancer center’s outpatient department. A convenience sampling strategy was used to recruit patients who were accessible during routine outpatient visits and willing to share their lived experiences. Additionally, a liver cancer specialist and the center’s head nurse, who were informed about the study’s purpose and methods, recommended participants capable of describing their experiences of FCR.

Participants voluntarily contacted the researcher in response to the notices or medical team referrals. After the study purpose and data collection process were thoroughly explained, participants provided informed consent and engaged in in-depth interviews. The study received approval from the Institutional Review Board (Approval No. 2019-07-073). Participants were informed of their right to pause or reschedule interviews if they experienced psychological distress; however, none opted to do so.

A total of 13 participants were enrolled in the study, with 11 (84.6%) being male. Ages ranged from 38 to 66 years, with seven participants (53.8%) in their 50s. Stage I was the most common diagnosis (46.2%). Among the six participants initially diagnosed at Stage I, four had experienced recurrence. Treatments included surgery and transarterial chemoembolization, and the time since diagnosis ranged from 2 to 126 months. Initially, inclusion criteria specified patients without recurrence, but due to the high recurrence rate, participants were included regardless of recurrence history. Among them, seven had experienced recurrence, while six had not (Table 1).

### 2.2. Preparation of the Researcher Team

The researchers brought extensive clinical and academic expertise. Rather than an interdisciplinary team, the researchers formed an intra-disciplinary team of oncology nurses with complementary areas of specialization. Both were certified oncology nurses with oncology ward experience and formal qualitative research training. The first author, K.R. Bae, with over 20 years of experience counseling oncology patients, led studies on the psychological challenges faced by patients with cancer and their caregivers. The second author, E. Jo, specialized in symptom management, quality of life, and quantitative FCR research. These complementary but discipline-specific backgrounds provided a robust intra-disciplinary foundation for rigorous data collection and analysis.

### 2.3. Data Collection

Data were collected through one-on-one in-depth interviews using a semi-structured questionnaire developed from a literature review and expert consultations. The interviews lasted approximately 50 min, were scheduled to coincide with the participants’ hospital visits, and were held in a private consultation room to ensure a comfortable and confidential setting.

Before each interview, participants were briefed on the study’s purpose and the audio recording process. During interviews, the researcher documented verbal and non-verbal cues and posed additional questions to elicit deeper insights. Interviews were transcribed promptly, incorporating contextual and emotional nuances.

### 2.4. Data Analysis

Data analysis was conducted using Colaizzi’s phenomenological method [25]. This approach involves extracting significant statements, formulating meanings, clustering these into themes, and synthesizing an exhaustive description to capture the essence of the phenomenon. The themes were cross-checked against the transcripts for accuracy, and external reviewers validated the findings. Theoretical saturation was confirmed when no new conceptual insights emerged, consistent with recent methodological guidance [26]. However, in conducting the analysis, we remained attentive to potential differences between fears of recurrence and fears of progression, particularly among participants with confirmed disease activity, in order to ensure that these nuances informed our interpretation.

### 2.5. Rigor of the Qualitative Research

To ensure rigor, this study applied Lincoln and Guba’s criteria [27] of credibility, transferability, dependability, and confirmability. Credibility was established by carefully selecting participants capable of articulating their FCR experiences, conducting semi-structured interviews with follow-up questions to enrich the data, and validating the findings through peer review. Although participant validation (member checking) was not performed due to concerns about imposing additional emotional burden on this clinically sensitive population, credibility was ensured through rigorous peer debriefing and external expert review. Transferability was ensured by continuing data collection until saturation was reached and including vivid direct quotations to contextualize the findings. Dependability was achieved by strictly adhering to Colaizzi’s method during the analysis, which qualitative research experts externally reviewed to ensure consistency and reliability. Finally, confirmability was maintained by employing bracketing techniques to minimize bias, thereby ensuring objectivity throughout the analysis. In addition, to minimize the influence of the researchers’ long-standing clinical experience, bracketing was reinforced through reflexive journaling, documentation of preconceptions prior to analysis, and regular peer debriefing sessions, ensuring analytic neutrality. This comprehensive approach underscores the trustworthiness and applicability of the findings in understanding the FCR experiences of patients with liver cancer.

## 3. Results

The analysis revealed that the experiences of patients with liver cancer with FCR comprised 13 themes, which were grouped into four theme clusters (Table 2) as follows: the inevitable reality of recurrence, amplified fears, changes in daily life driven by fear, and living with fear.

### 3.1. Theme Cluster 1: The Inevitable Reality of Recurrence

Participants perceived liver cancer recurrence as an inevitable outcome. They attributed this to either limitations in self-care or the natural progression of their condition. This dual acknowledgment—accepting recurrence while recognizing the improbability of complete recovery—was a shared experience among participants. Although this perception was shared across participants, those with a history of recurrence experienced this inevitability as an immediate and lived reality, whereas participants without recurrence described it as a potential future event rather than a present threat.

1. Acceptance of the inevitability of recurrence

The participants stated that they were informed about recurrence by their medical team and were aware of other patients with liver cancer who had experienced recurrence. Both those who had and those who had not experienced recurrence mentioned the inevitability of facing it at some point, reflecting an attitude of calm acceptance.

*Recurrence...doctors and people I know say that no matter how well you manage it, the limit is 10 years. That kind of pressure was there. Liver cancer doesn’t use the word ‘cure,’ so I’ve always kept recurrence in mind.*

(Participant 5)

*Honestly, if it happens again, I don’t think much about it. I just think, well, it will come when the time comes.*

(Participant 11)

2. Self-blame for insufficient self-care

Some participants perceived recurrence as a natural consequence of their insufficient self-care, such as excessive alcohol consumption or overworking. They accepted their diagnosis and recurrence as inevitable outcomes of their past choices.

*I see this as an opportunity. Because I feel like I’ve mistreated my body too much, and I need to reflect on it. Drinking, overworking, I brought all this on myself.*

(Participant 9)

3. Disappointment despite best efforts

The recurrence of liver cancer deeply disheartened participants who had meticulously managed their health following their cancer diagnosis. Although they had mentally prepared for the possibility, they were overwhelmed by the realization that their diligent efforts had ultimately been in vain.

*I really avoided drinking. Of course, I’ve been working a lot in my job, but it’s not like I’ve been doing any physically demanding labor. And then, I had been avoiding all the foods I was told not to eat, sticking to my diet, and yet, it came back. This is what they mean by panic. I lived well for five years, and now this. I couldn’t see anything around me.*

(Participant 7)

### 3.2. Theme Cluster 2: Amplified Fears

Despite outward acceptance of recurrence, participants experienced heightened physical and psychological fears, especially those with prior recurrence.

4. Recurrent physical and emotional anxiety every three months

Regular follow-ups every three months, including imaging and tumor marker tests, were a major source of stress, particularly in the initial years post-treatment. Participants reported severe anxiety and physical symptoms leading up to these tests, which temporarily eased after receiving favorable results.

*Normally, I don’t feel anything particular, but a week before the checkup, even minor aches make me wonder whether something has developed over the past three months. Then I feel helpless. The night before the checkup, I can barely sleep, and when I do, I have strange dreams. Then, for a week after the checkup, it gets even worse. It’s like the pain gets even more painful. But after meeting with my doctor, all the pain disappears.*

(Participant 7)

*We get checkups every three months, right? CT scans and all that—it feels like life happens in three-month intervals. That’s how it feels. The closer I get to running out of the medication from the hospital, the more nervous I feel. I wonder if it’s gotten worse. It takes about a week to get CT results, and during that week, so many thoughts run through my mind.*

(Participant 13)

5. Amplified fears from cases encountered in others

Participants’ fears of recurrence were reignited by media reports, online content, or hearing about others’ cancer experiences. Stories of diagnosis, treatment, recurrence, or death often heightened their anxiety, leading them to contemplate their own vulnerability.

*When I watch YouTube videos, I can’t help but notice people with the same type of cancer. I hear things like, ‘If you’re not careful, even in early stages, it can come back,’ and it makes me realize how frightening cancer really is.*

(Participant 9)

*When I see something on the Internet or TV about cancer, even if it’s unrelated to me, I think, ‘Oh? Could that happen to me too?’ Things I wasn’t thinking about come back to mind—it makes me think again, even if I had forgotten for a while.*

(Participant 10)

6. Intensified fear after recurrence

The participants who had experienced recurrence reported significantly heightened fears compared to their initial diagnosis. While the fear of recurrence tends to diminish gradually following treatment, the participants noted that it resurged with greater intensity after each recurrence, often reaching new peaks. Even minor health issues triggered a renewed fear of recurrence.

*I would say it’s gotten worse. If my fear was at level 1 before, it’s now at 1.5 to 2. I get this sudden influx of fear that if I let my body become ever so slightly unwell or tired, it will hit me again.*

(Participant 5)

*On a scale of 10, at first, it was at 6. Over time, as nothing happened, it gradually went down to 5, 4, 3, 2, and 1. But then it came back, and the intensity shot up to 9. Then it happened again, and from then on, it’s at 10.*

(Participant 7)

### 3.3. Theme Cluster 3: Changes in Daily Life Driven by Fear

Fear of recurrence led to significant lifestyle changes among participants, particularly those of working age. Many faced difficulties returning to work or had to lower their career goals due to health concerns. Uncertainty about recurrence made future planning challenging, while reduced social interactions contributed to feelings of isolation and alienation.

7. Lowered life goals

Participants reduced their career aspirations due to health concerns following their liver cancer diagnosis. To prioritize their well-being, they avoided overworking and social gatherings involving alcohol, which limited opportunities for professional advancement and networking.

*I adjusted my ambitions. I decided not to aim for a director position. My body and liver can’t handle the stress along the way. I’m now handicapped when it comes to human networking through man-to-man talking without reservation. That’s men’s world, where drinking is still essential for bonding even if you’re not a heavy drinker. So, I lowered my goals.*

(Participant 7)

*I can’t work as boldly as before, which brings its own anxieties. Returning to work has become a bit more difficult for that matter.*

(Participant 10)

8. Anxiety about an uncertain future

Participants struggled with planning for the future due to the unpredictability of recurrence. Younger participants, particularly those with families, were anxious about providing for their dependents, while long-term goals like retirement planning felt out of reach.

*Now, there’s just so much to worry about that makes me hesitate, not daring to act. Yet, the kids are still growing, and my wife is still young, so I need to earn a living to support them. There’s no way I can plan for retirement now—that’s my main concern.*

(Participant 5)

*I’m just living my life right now without any plans. Managing my life is possible only when it is guaranteed that the next five years will be without recurrence. I haven’t really made plans about what to do and how to live. I know I should start thinking about it since it’s been two years, but I don’t have any clear idea about that.*

(Participant 1)

9. Social isolation and alienation

Participants reported feeling isolated as they avoided social gatherings, especially those involving alcohol, due to fears of recurrence. Recurrence-related treatments further limited their social interactions, leading to a restricted social life.

*My social relationships aren’t what they used to be. I’m more afraid of recurrence now, and in our culture, gatherings automatically involve drinking. I avoid those situations as much as I can, and if I do go, I leave early.*

(Participant 10)

*These days, I feel that having a long battle with liver cancer makes me feel increasingly lonely and isolated. The calls I used to receive ten times a day gradually decreased, and I feel more and more disconnected from society.*

(Participant 13)

### 3.4. Theme Cluster 4: Living with Fear

Participants accepted the fear of recurrence as a constant in their lives, viewing it as something to live with rather than eliminate. They remained hopeful, relying on medical guidance and optimism about future advancements to navigate their journey.

10. Acceptance of reality as it is

Participants acknowledged the inevitability of their fear of recurrence and focused on coexisting with it constructively, rather than trying to suppress or eliminate it.

*If I have to carry it with me till the end, I’d rather pacify it instead of trying to get rid of it. I said to myself, ‘Don’t try too hard to put it to sleep; just find positive and enjoyable patterns for your life.*

(Participant 7)

*Ah, this is what it’s like to live with it, fixing parts as they break, relying on medical advancements to keep moving forward. That’s the way to go.*

(Participant 12)

11. Efforts to remain relaxed and unconcerned

Despite the challenges of fully accepting their fear, participants made conscious efforts to stay relaxed and avoid overthinking.

*At first, I couldn’t eat properly. Not only that, if someone complained about a food item on the table, I’d suddenly feel pain just eating it, even though I was enjoying eating it. But after being told not to worry and just live at ease, I started working and moving again. Then it faded out gradually.*

(Participant 8)

*I try to lead a simple life. Thinking too much doesn’t help at all, so I try not to stress and just let things be.*

(Participant 13)

12. Trust in medical guidance

The participants believed that the best way to prevent recurrence was to prioritize their health by strictly following medical advice. They avoided complementary and alternative therapies, which they felt were unsuitable for patients with liver cancer, and placed their trust in their doctors’ guidance.

*I look up to my doctor like a god. I figure if I follow their instructions to the letter, I might be able to extend my life by another decade or so. That’s why I’ve given up drinking, smoking, and other old habits, and I’m diligent about taking my medications.*

(Participant 4)

*‘Don’t drink alcohol.’ ‘No, I won’t drink.’ ‘Don’t eat raw food.’ ‘No, I won’t eat it.’ ‘Eat well.’ ‘Yes, I’m eating well.’ I follow all the instructions and exercise diligently. There’s nothing else I can do, is there?*

(Participant 7)

13. Hope for new treatments

Participants found encouragement in advancements like immunotherapy, maintaining hope that future treatments could help them manage liver cancer recurrence and reduce their fear.

*I guess my liver is acting out because of all the strain I’ve put on it. But I think, Medicine keeps improving—someday, there’ll be something to get rid of all this.*

(Participant 7)

*Recurrence is inevitable, and I live with constant tension. But I have to accept it. There’s not much I can do, but when my doctor says there’s a new, better drug and asks me to take it, that’s the best thing I could hear.*

(Participant 13)

## 4. Discussion

This study explored the in-depth experiences of patients with liver cancer regarding their FCR through one-on-one in-depth interviews. The FCR-related experiences are discussed below, organized into the four theme clusters identified during the analysis of the interview transcripts.

First, participants often perceived recurrence as inevitable due to liver cancer’s high recurrence rate and their past experiences. This perception fostered resignation and acceptance, with many acknowledging it could happen any time. Similar patterns are seen in patients with other high-recurrence cancers like ovarian and breast cancer, though patients with liver cancer face additional challenges due to underlying chronic liver conditions. Perceived inevitability of recurrence can hinder proactive health management, occasionally leading to neglect of care [28]. To counter this, collaborative efforts between patients and healthcare providers are essential. Setting realistic goals, creating actionable plans, and emphasizing patient responsibility in care can encourage engagement [29]. Aggressive antiviral treatments significantly reduce recurrence risk, highlighting the need for consistent adherence to treatment plans and informed guidance from healthcare providers. Constructively addressing uncertainty about cancer progression is also vital. This involves clear explanations of symptoms, fostering trust-based communication, and emphasizing preventive care [30]. Tailored psychosocial interventions can help mitigate fear of recurrence, improve quality of life, and sustain health management. Future research should explore how such interventions can be customized to address the unique needs of patients with liver cancer.

Second, participants experienced heightened emotional distress when thoughts of recurrence emerged. This persistent fear contributed to psychological challenges, including despair, reliance on spirituality, post-traumatic stress, and uncertainty about health and the future [14]. Research on high-recurrence cancers, such as ovarian and lung cancers, highlights the exacerbation of psychological burdens due to FCR and its negative impact on treatment adherence and outcomes, suggesting parallels in patients with liver cancer [31,32]. In our findings, patients with previous recurrence described significantly more intense and persistent fear responses—particularly during follow-up periods—compared with those without recurrence. These individuals reported escalations in fear intensity after each recurrence event, indicating that recurrence fundamentally reshapes the emotional trajectory of FCR. In contrast, participants without recurrence experienced more intermittent worries and generally returned to periods of psychological stability. This distinction strengthens the clinical relevance of our findings and underscores the need for recurrence-specific psychological support. Patients with recurrence reported more intense FCR, amplifying their emotional distress and emphasizing the urgent need for effective psychological support [33,34]. Recent studies propose web-based interventions as accessible, cost-effective tools for managing FCR. These interventions offer immediate support compared to traditional counseling; however, additional evidence is required to confirm their efficacy and optimize their implementation [35,36]. Practical strategies, including pre-screening education, emotional support, and stress management techniques such as regular exercise and meditation, can empower patients to regain control and enhance resilience. Recognizing FCR as a multidimensional issue with significant implications for quality of life underscores the need for tailored interventions. Addressing this fear holistically can alleviate emotional distress, improve psychological stability, and promote better health outcomes for patients with liver cancer [37].

The third theme cluster, “changes in daily life driven by fear,” highlighted the profound impact of FCR on participants’ social and personal lives, with some reporting lowering their social and career aspirations, facing uncertainty about future planning, and experiencing significant social isolation and withdrawal. This withdrawal was often driven by feelings of being a burden to others and the stigma associated with liver cancer and chronic liver conditions, which exacerbated psychological stress and further reduced their social quality of life [38,39,40]. In this context, self-blame and social withdrawal were closely intertwined with stigma, as many participants associated their illness with socially disapproved behaviors such as alcohol consumption or overwork, leading to heightened shame and concealment. In particular, shame and internalized stigma related to alcohol use or chronic liver disease intensified both self-blame and social withdrawal, compounding the psychological burden beyond FCR alone. Social support emerged as a critical buffer against these challenges, helping to alleviate psychological distress, promote emotional stability, and encourage positive coping strategies. Support from family, friends, and colleagues, combined with empathetic guidance from medical teams, can empower patients to accept their circumstances and foster resilience. Encouraging open discussions about FCR and normalizing these fears within supportive environments are key strategies for intervention [41]. Additionally, these feelings of shame and self-blame appeared to be reinforced within Korea’s alcohol-centered social culture, which several participants described as intensifying their tendency to withdraw from social interactions. Collaborative efforts among family, friends, and healthcare providers can reduce FCR’s burden, enabling patients to regain control over their lives and plan for a more hopeful future.

The fourth theme cluster, “living with fear,” showed that patients with liver cancer chose to accept FCR as an integral part of their lives rather than attempting to suppress or eliminate it. This form of acceptance differs from the earlier, resignation-based acceptance described in Theme Cluster 1. At the same time, acceptance in this population may risk shifting into resignation, potentially reducing vigilance in self-care; thus, clinicians should remain attentive to whether acceptance reflects adaptive coping or passive disengagement. This allowed them to maintain daily routines and foster a positive outlook despite uncertainties about recurrence and metastasis. Similar findings have been observed in patients undergoing transarterial chemoembolization, who sustained positive attitudes and normal lives despite recurrence risks [24]. Importantly, coping strategies differed by recurrence history. Participants with recurrence developed more layered and effort-intensive approaches to coexisting with fear, reflecting the cumulative psychological impact of repeated fear activation. Conversely, those without recurrence tended to show steadier, less fluctuating adaptation patterns. These contrasting trajectories suggest that ‘living with fear’ is not a uniform process but rather one that is strongly influenced by recurrence status and prior disease experiences. Participants actively developed personalized coping strategies, demonstrating resilience and psychological stability even while living with persistent FCR. Trust in medical teams and adherence to treatment recommendations were pivotal survival strategies, enabling patients to transition from a passive to an active role in their care. Rather than merely following medical advice, patients participated in decision-making processes, leveraging their illness experiences to manage their quality of life more effectively [42]. This proactive approach contrasts with traditional patient roles, highlighting the need for tailored interventions. Medical teams can help patients with liver cancer embrace FCR constructively by understanding their illness perceptions and actively collaborating with families to provide timely, personalized support. Sharing advancements in treatments, integrating patient perspectives, and fostering trust through clear communication are critical. Collaborative decision-making that respects patient preferences and balances psychological and medical support can instill confidence and improve quality of life.

### 4.1. Implications

This study fills a critical gap in FCR research by exploring the unique psychological experiences of patients with liver cancer, a population often overlooked compared to those with breast or colorectal cancer. Owing to liver cancer’s high recurrence rate and uncertain prognosis, patients face distinct psychological challenges. By examining how they experience, accept, and adapt to FCR, this study provides foundational data for developing tailored psychological and psychosocial interventions. These findings also offer valuable insights for designing psychosocial support programs aimed at mitigating the impact of FCR on patients’ lives.

### 4.2. Limitations

A key limitation of this study is the inclusion of patients both with and without experience of recurrence. Recurrence profoundly affects physical, psychological, social, and spiritual contexts, which may influence FCR differently. While this study observed that patients with recurrence were more affected, both groups exhibited similar overarching experiences. Future research should focus on identifying and comparing the nuanced differences between these groups to enhance understanding and intervention development.

## 5. Conclusions

This study provides an in-depth understanding of FCR among patients with liver cancer, revealing significant psychological, social, and lifestyle changes driven by persistent fear due to the high recurrence rate and uncertain prognosis. While FCR intensity varied with recurrence status, it affected all patients, prompting them to integrate the fear into their lives and develop adaptive strategies. Although FCR negatively impacts quality of life, it also serves as a psychological motivator for positive coping. These findings highlight the need for psychosocial interventions to address FCR and improve the quality of life for patients with liver cancer.

## Figures and Tables

**Table 1:** General characteristics of the participants.

ID	Sex	Age	Stage	Cancer Treatment	Time Since Diagnosis (in Months)	Recurrence
1	F	44	I	OP, TACE	30	Y
2	M	66	II	TACE, PBT	15	N
3	M	60	I	RFA, TACE	126	Y
4	M	61	II	OP, RFA	21	Y
5	M	54	II	OP	37	Y
6	F	38	I	RFA	20	N
7	M	59	I	OP, RFA	116	Y
8	M	52	II	TARE	19	N
9	M	54	I	OP	2	N
10	M	50	III	OP	13	N
11	M	46	III	OP	14	N
12	M	55	II	OP, RFA, TACE, PBT	15	Y
13	M	50	I	TACE, PBT	27	Y

OP: operation; TACE: transarterial chemoembolization; PBT: proton beam therapy; RFA: radiofrequency ablation; TARE: transarterial radioembolization; Y: Yes; N: No.

**Table 2:** Theme clusters and themes related to the FCR experience among patients with liver cancer.

Theme Cluster	Theme
Inevitable Reality of Recurrence	– Acceptance of the inevitability of recurrence – Self-blame for insufficient self-care – Disappointment despite best efforts
Amplified Fears	– Recurrent physical and emotional anxiety every three months – Amplified fears due to cases encountered in others – Heightened fear following recurrence
Changes in Daily Life Driven by Fear	– Lowered life goals – Anxiety about an uncertain future – Social isolation and alienation
Living with Fear	– Acceptance of reality as it is – Efforts to stay relaxed and unconcerned – Trust in medical guidance – Hope for new treatment methods

## Data Availability

The data underlying this article will be shared on reasonable request to the corresponding author. Data sharing is subject to ethical considerations and institutional guidelines to ensure the privacy and confidentiality of participants.

## Abbreviations

The following abbreviations are used in this manuscript:

FCR	Fear of cancer recurrence
